# Protective Immunity Based on the Conserved Hemagglutinin Stalk Domain and Its Prospects for Universal Influenza Vaccine Development

**DOI:** 10.1155/2014/546274

**Published:** 2014-05-25

**Authors:** Madhu Khanna, Sachin Sharma, Binod Kumar, Roopali Rajput

**Affiliations:** Department of Respiratory Virology, Vallabhbhai Patel Chest Institute, University of Delhi, Delhi 110007, India

## Abstract

Influenza virus surface glycoprotein hemagglutinin (HA) is an excellent and chief target that *elicits* neutralizing antibodies during vaccination or natural infection. Its HA2 subunit (stem domain) is most conserved as compared to HA1 subunit (globular head domain). Current influenza vaccine relies on globular head domain that provides protection only against the homologous vaccine strains, rarely provides cross-protection against divergent strains, and needs to be updated annually. There is an urge for a truly universal vaccine that provides broad cross-protection against different subtype influenza A viruses along with influenza B viruses and need not be updated annually. Antibodies against the stem domain of hemagglutinin (HA) are able to neutralize a wide spectrum of influenza virus strains and subtypes. These stem-specific antibodies have great potential for the development of universal vaccine against influenza viruses. In this review, we have discussed the stem-specific cross-reactive antibodies and heterosubtypic protection provided by them. We have also discussed their epitope-based DNA vaccine and their future prospects in this scenario.

## 1. Introduction

Influenza virus belongs to Orthomyxoviridae family and is lipid enveloped with negative sense single stranded RNA segmented genome. The envelope of the virion contains two surface glycoproteins, hemagglutinin (HA) and neuraminidase (NA), which are responsible for virus entry via attachment to the host cell sialic-acid receptors and progeny release, respectively. Three types of influenza viruses, namely, influenza A, B, and C type viruses, are based on the unique identity of the internal proteins, the nucleoprotein (NP), and the matrix (M1) protein. Influenza A virus is further divided into various subtypes, on the basis of two surface glycoproteins, HA and NA. Influenza A virus has 18 different HA and 11 different NA surface glycoproteins [1]. These different HA are divided into two phylogenetic groups: group 1 [H1, H2, H5, H6, H8, H9, H11, H12, H13, H16, and H17] and group 2 [H3, H4, H7, H10, H14, and H15] on the basis of their nucleotide sequences [2].

Influenza A virus, apart from humans, infects a variety of animals such as pigs, horses, sea mammals, and birds, whereas influenza B and C types mostly infect human beings. Influenza A virus experiences two major antigenic changes, antigenic shift (gene reassortment) and antigenic drift (point mutation), whereas influenza B and C viruses undergo antigenic drift only. Antigenic drift in viral genome is responsible for emergence of new strains that cause seasonal epidemics. Occasionally, antigenic shift leads to emergence of novel strains that are immunologically naive to population through gene reassortment and cause influenza pandemic. Influenza A viruses are responsible for both pandemic and seasonal epidemics, while influenza B and C viruses only cause epidemics. The last century witnessed three pandemics: Spanish flu (1918, H1N1), Asian flu (1957, H2N2), and the Hong Kong flu (1968, H3N2). The 1918 Spanish flu killed estimated 50–100 million people worldwide, while the Asian flu and the Hong Kong flu pandemics claimed approximately 500,000–2,000,000 human lives [3, 4]. In the beginning of the 21st century, in April 2009, another pandemic occurred due to novel swine influenza H1N1 that spread worldwide across 214 countries and caused 500,000–1,000,000 deaths [5]. Each year, seasonal influenza virus infects 100 million people worldwide causing three to five million severe infections and approximately 500,000 deaths [6]. The morbidity and mortality due to influenza virus infection need to be managed efficiently for the sake of public health. Vaccination is the most effective way to protect population from influenza virus; this can reduce the impact of epidemic as well as pandemic influenza. The current WHO recommended vaccine being used needs to be updated annually. These vaccines provide protection only against the homologous vaccine strains and closely related variants and rarely provide cross-protection against divergent strains within and across the subtype [7].

H1, H2, and H3 are the major subtypes of influenza A viruses that infect human beings, while occasional sporadic infections of H5, H7, or H9 subtypes have also been reported [8–10]. Human infection of avian H5N1 subtype has been increasing in the last decade, and, recently, avian origin H7N9 human infection in China is considered a major potential threat for a future influenza pandemic [11, 12]. There is an urge for a truly universal vaccine that covers all subtypes of influenza A viruses and both lineages of influenza B viruses, so that it provides protection against heterosubtypes of influenza A viruses along with influenza B viruses. These universal vaccines provide broad cross-protection and need not be updated annually. In this review, we have focused on the stem of hemagglutinin- (HA-) specific antibodies that can provide cross-protection against different subtypes of influenza A and influenza B viruses. We have also addressed the future prospects of their epitope-based DNA vaccine.

## 2. Hemagglutinin (HA) Surface Glycoprotein

The fourth segment of viral genome encodes hemagglutinin, the surface glycoprotein. It plays a vital role in the attachment and activation of membrane fusion for entry of the virus into host cells. Viral hemagglutinin precursor (HA0) is cleaved at arginine amino acid residue (rarely lysine) by host trypsin-like proteolytic enzymes such as plasmin and tryptase found at respiratory and gastrointestinal tracts epithelial cells, into HA1 and HA2 subunits. These subunits form spikes which protrude externally [13]. HA2 subunit is highly conserved as compared to HA1 subunit [14]. Each monomer of HA molecule can be distinguished into a globular head domain and stem (stalk) domain. The globular head domain is part of HA1 subunit that contains receptor binding region, whereas stem domain is part of both HA1 and HA2 subunits that are responsible for fusion of host endosomal membrane with the viral membrane, to release genetic content (ribonucleoprotein) into the host cell. During natural infection, virus neutralizing antibodies are generated predominantly against the globular head domain, especially to antigenic region that surrounds the receptor binding pocket, and less abundantly against the stem domain which is comparatively conserved. Globular head domain antibodies prevent viral binding to the host cell receptor, whereas an anti-stem antibody prevents the fusion step of viral entry. Antibodies recognizing globular head neutralize the virus and provide good protection against infection but do not cross-react with the HA of other subtypes [15]. The anti-stem-specific antibodies are highly cross-reactive with phylogenetically related, but rarely phylogenetically distinct HA [16, 17]. These anti-stem-specific antibodies can be prime candidates of universal vaccine development that can provide cross-protection against different subtype influenza A viruses along with influenza B viruses.

## 3. Stem-Specific Antibodies and Cross-Reactivity

The presence of well conserved HA antigenic site cross-protection was first reported on two distinct subtypes, H1 and H2, at HA1 subunit C-terminus (aa 318–322) and HA2 subunit N-terminus (aa 47–58) that could be recognized by mAb C179 which neutralized all of H1 and H2 strains but not H3 strains [18]. In another study, it was observed that mAb CF2, specific to HA2 conserved region particularly N-terminus (aa 1–38), provides intrasubtype cross-protection in mice against lethal infection. These antibodies, targeted to conserved region, do not neutralize the viral infectivity, but they only inhibit the fusion activity of influenza viruses that reduces virus replication and mediate effective recovery from infection in mice against homologous strain and heterologous strain within the same subtype [19]. In an experiment, the mice immunized with recombinant vaccinia virus (rVV) expressing HA2 of subtype, homologous to virus challenges, did not prevent virus infection but accrued the mice survival and faster elimination of virus from lungs. Passive immunization with purified antibodies from rVV confirms the described effects on virus infection [15]. Ekiert et al. (2009) showed that the broadly neutralizing human antibody CR6261 recognizes a highly conserved helical region in the membrane-proximal stem of HA1 and HA2 and identified these epitopes as target region to design improved vaccines that can elicit CR6261-like antibodies and which can be used as antibody-based therapies for the treatment of influenza [7]. The replacement of seasonal strains is mediated by a population-scale boost in antibodies specific for conserved regions of the hemagglutinin stalk. Pica et al. (2012) prove this by developing chimeric hemagglutinin of H3 globular head and H1 stem region to generate the stem-specific antibodies in humans. It shows that infection with 2009 pandemic H1N1 virus elites the anti-stem (stalk) antibodies in population that gets rid of the circulating seasonal H1N1 from human population. Similarly, during 1968 Hong Kong flu, pandemic H3N2 virus overlooked the circulating H2N2 from population by generating antibodies specific to N2 subtype neuraminidase. They also found that these stalk-specific antibodies* showed* reactivity with H5 HA subtype. H5 HA subtype and H1 HA subtype share very similar structures and belong to the same phylogenetic group [20].

The globular head domain of the intact HA molecule inhibits recognition of stem region by immune cells, due to steric shielding or immune dominance of the membrane distal portion of HA protein. Researchers have vaccinated mice with DNA vaccine followed by booster virus like particle encoding headless HA of influenza A/Puerto Rico/8/34 (PR8). It induced the antibodies which are cross-reactive among group 1 HA subtype, whereas full length PR8 vaccine did not show any antibody response [21]. In another study, Eggink et al. (2014) modulated the immune response towards conserved stalk domain by hyperglycosylation of globular head domain which masked the immunodominant region. It induced higher titer of stalk-specific antibodies that provided broad cross-protection as compared to the wild type hemagglutinin. The full length HA was incompetent to induce stem-specific antibodies [22]. Wang et al. (2010) vaccinated the mice from synthetic peptide conjugate vaccine of long *α* helix (LAH) coupled with keyhole limpet hemocyanin (KLH). LAH is the highly conserved region of HA2 of hemagglutinin protein of H3 and other subtypes which elite neutralizing antibodies (nAb) 12D1 that bind at 76–130 amino acids residues. It proved efficacious in protection against H3 virus subtype and moderately against other subtypes, for example, H5, H7, H2, and H1. This LAH-KLH vaccination boosted serum IgG and IgM with indicated T-cell dependent antibody production that suggests affinity maturation [23]. In another study, researchers have found that the HA2- (stem) based antibodies provide cross-protection against several strains within a subtype but not to different phylogenetic group subtypes. Bommakanti et al. (2010) constructed HA2-based recombinant immunogen from H1N1 A/Puerto Rico/8/34 and drifted strains of A/New Caledonia/20/99 and A/California/07/09 and expressed in* E. coli*. Immunization with these purified recombinant stem domains protected the mice from lethal challenge by A/PR/8/34. In a separate experiment, they found that the stem domain derived from A/Hong Kong/68 (H3N2) failed to protect mice from A/Puerto Rico/8/34 (H1N1) virus challenge. This shows that stem domain immunogen can protect against several strains of viruses within the subtype unlike conventional vaccines that are ineffective against drifted virus strain, but it failed to protect against different phylogenetic group subtypes [24]. Stanekova et al. (2012) investigated HA2 antibody generation in humans during natural infection and identified their epitope specificity and found more antibodies specific to HA2 residue 125–175 recognized by mAb IIF4 than mAb FC12 on this region. And poorly recognized by mAb CF2, epitope located in-between 23–38 residues of HA2. It suggests that HA2-based antibodies are generated during natural infection, although the titer of these antibodies is low. Even after vaccination, there is negligible increase in the titer of HA2-specific antibodies. Thus, enhancement of these HA2-based antibodies could contribute to prevention and reduce severity of influenza infection in humans [25]. To increase titers of stem (stalk) directed antibodies, Krammer et al. (2013) repeatedly immunized mice with constructed chimeric HA (cHA) that expressed the same stalk of H1 and irreverent head of H5, H6. These chimeric constructs induced broadly neutralizing stalk-specific antibodies and protected mice against challenge of H5N1, H6N1, pH1N1, and H1N1 (A/Puerto Rico/8/34). This study demonstrated the efficacy of cHA constructs in eliciting broad-spectrum immunity against group 1 HA of influenza viruses [26]. Similar approach was also used to generate group 2 HA stalk-specific antibodies using stalk of H3, and it provided protection against H3, H10, H14, H15, and H7 [27]. Throsby et al. (2008) constructed human monoclonal antibody libraries from IgM+ memory B-cell from donor and unique combination of VH genes (variable heavy) that were selected using strong affinity to different H5 rHA antigen. Of the total combinations, the mAb CR6261 was the most potent neutralizing antibodies. Both mAb CR6261 and CR6323 showed strong reactivity to rH1, rH5, and rH9 but not to rH3, rH7, or rHA of influenza B viruses. The epitopes of CR6261 and CR6323 cross-neutralizing mAb localized in the stem domain of HA [28]. Thus, broadly neutralizing antibodies such as CR6261, F10, and C179 bind to HA proteins from group1, but they do not bind to HA proteins of group 2 (neutralized by mAb 12D1 and CR8020); hence, they do not neutralize viruses of their group, possibly because of glycosylation at the residue of Asp 38 of HA1 in A/HK/68 and the other group 2 HAs (H7, H10, and H15) that is absent in group 1 HAs [24]. Corti et al. (2011) are able to isolate FI6 neutralizing monoclonal antibody that recognized and neutralized both group 1 and group 2 hemagglutinin (HA) surface glycoproteins from human plasma B-cell. The conserved epitope recognized by FI6 is found in F subdomain of HA [17], whereas Cyrille Dreyfus et al. (2012) found that the CR9114 recognizes the conserved epitope in the hemagglutinin (HA) stem and neutralizes both influenza A and B viruses. They also found CR8033 and CR8071 human monoclonal antibodies in the study that are able to recognize distinct conserved epitope present in globular head region of the influenza B hemagglutinin (HA) [29]. Lee et al. (2013) investigated the antibodies and cell-mediated responses against recombinant H5 HA2 protein (residues 15–137) and observed a long term immunity. They found that it provided 100% protection from mortality against the same subtype virus challenges and 80% protection against different subtypes within the same phylogenetic group. These rH5 HA2 proteins are capable of inducing Th1 and Th2 type cellular immune response and the immunity was found to be maintained up to 6 months after the last immunization [30]. Stanekova et al. (2013) fused the HA2 segment (residues 23–185) of H3 with genetically detoxified adenylate cyclase toxoid (CyaA-E5) to increase the immunogenicity and to develop a novel strategy for antigen delivery to antigen presenting cells (APCs). It induced HA2_93–102,_ HA_296–104,_ and HA2_170–178_ stem-specific antibodies and CD4^+^ helper, as well as cytotoxic CD8^+^ T lymphocyte (CTL) responses. The immunized mice were cross-protected against the lethal challenge dose of a homologous virus (H3 subtype), as well as against the infection with a heterologous (H7 subtype) influenza A virus. These HA2-specific antibodies showed cross-reactivity within group 2 HA proteins (H3, H4, and H7); however, their reactivity within subtypes of H1 belonging to group 1 was weak. The authors presented the first report on heterosubtypic protection against influenza A virus infection mediated by an HA2- (stem) based vaccine that induced both humoral and cellular immune responses without the need of an adjuvant. This finding suggests that the HA2-based vaccine can elite both humoral and cell-mediated immune responses and may be proven to be a novel vaccine strategy [31]. DiLillo et al. (2014), in an* in vivo *study, showed that stalk-specific antibodies are Fc*γ*R interaction dependent for the maximum neutralization of influenza virus and induce the antibody dependent cellular cytotoxicity (ADCC) [32]. These studies support HA2 subunit vaccine as candidate that harbors great potential for production of universal vaccine against influenza viruses.

## 4. Stem Epitope-Based Universal DNA Vaccine

Vaccination is the primary strategy for prevention and control of influenza. The ideal vaccine should induce good humoral and cellular immune response to efficiently reduce morbidity and mortality. Conserved epitopes from the influenza nucleoprotein (NP), matrix (M1 and M2), and HA proteins are the major targets in the search for a universal vaccine. The development of a universal influenza vaccine will need elicitation of antibodies against the conserved HA stem region having the ability to cross-react with HA proteins within the influenza virus types and subtypes. These universal vaccines, based on the conservation of the stalk (stem) domain of the HA, provide broad protection against all circulating human influenza virus strains as well as potential pandemic subtypes.

DNA vaccines have great potential as an alternative conventional vaccine capable of inducing protective immune responses against a variety of infectious diseases. Wolff et al., in 1990, for the first time demonstrated that plasmid DNA vaccine can be expressed upon direct inoculation into mouse muscle [33]. These DNA vaccines can encode recombinant DNA molecule of multiple antigen gene, which reduces manufacturing cost and time as compared to the conventional vaccine that we are currently using, without carrying infections that are associated with live attenuated vaccines. These plasmid-based DNA vaccines are able to express high levels of proteins of interest in cells and can induce both humoral and cellular immune responses. They can also override maternal antibodies [34].

Our current vaccine strategy against influenza virus is based on surface antigen-directed antibody responses and avoiding cellular immune responses. These antibody responses are highly specific and so susceptible to evasion by antigenic drift and shift. Cellular immune responses are far more cross-reactive. The CD4^+^ and CD8 T^+^ cell receptors would allow them to recognize epitopes even after the addition of a point mutation [35]. Plasmid DNA under the control of a strong eukaryotic promoter can be used for immunization by injection into the host cell (e.g., myocytes or dendritic cells). After the processing of these proteins, immunogenic peptides will be presented by MHC class I molecules to virus-specific T cells [36].

Dai et al. (2013) constructed DNA vaccine expressing a conserved region of the HA protein linked with Ag85A protein that gave protection against both influenza and secondary infection with* Staphylococcus aureus* (*S. aureus*). Secondary pneumonia infection due to* S. aureus *is a significant cause of mortality associated with influenza A virus (IAV) infection.* M. tuberculosis* secreted antigen Ag85A may serve as a good immune adjuvant for HA2 and induces Th1 type response in lungs and splenocytes of inoculated mice. It increases survival after IAV infection and reduced bacterial load after* S. aureus* challenge which was associated with vaccine-induced TLR2 expression [37]. This study shows that we can develop two or more epitope-based recombinant DNA molecule vaccines. These fused epitope DNA vaccines can provide protection against primary and secondary infections simultaneously. They generate not only humoral immune responses but also cell-mediated immune responses against our targeted antigen by simply injecting DNA plasmid into host via intramuscular or intranasal routes and in antigen presenting cell.

## 5. Traditional Influenza Vaccines and Their Approach

Currently, trivalent and quadrivalent inactivated split or live attenuated split vaccines have been recommended. Trivalent vaccine contains influenza A/H1N1 pdm09, A/H3N2, and B strains, whereas quadrivalent vaccine contains two influenza B virus strains considered to be the most likely to circulate in the upcoming influenza season in addition to influenza A virus strains. Influenza vaccine is formulated to contain the viruses or their HA proteins. These vaccines rely on surface HA antigen directed antibody responses and ignore cellular aspects to prevent infection. Inactivated influenza vaccine production begins with the generation of hybrid vaccine reference strain with the HA and NA genes from the drifted variant combined with other genes from a laboratory strain adapted to grow well in eggs [38]. These harvested virions are then chemically inactivated by formaldehyde or *β*-propiolactone, the viral envelope is disrupted with detergents, and the HA and NA proteins are then purified to be used as the main immunogens in inactivated influenza vaccine. Live attenuated influenza vaccines (LAIV) are produced through genetic reassortment between a donor virus and the wild type strain of influenza virus in tissue culture yielding an MVS (master virus strain). The MVS now contains two genetic segments that encode for HA and NA derived from the antigenically relevant influenza viruses recommended for inclusion in the annual vaccine and the remaining internal segments from the attenuated donor strain. These vaccines provide strain-specific protection and must be updated annually. They are based on good match between circulating strains which are selected by global surveillance on influenza virus and isolates included in the vaccine. WHO selection committee selects vaccine strains approximately 6 to 9 months before the onset of influenza season, thus allowing sufficient time to vaccine manufacturers and distributors to develop influenza vaccines. Influenza viruses are constantly undergoing change every 3 to 5 years, where the predominant strain is replaced by another variant strain that evades the existing antibody responses [39].

Inaccurate prediction in selection of strain for vaccine and significant changes in viruses reduce the efficacy of vaccine [40]. These vaccines provide protection against homologous vaccine strain and closely related variants and rarely provide cross-protection against divergent strains within and across subtypes.

## 6. Benefit of Stem Epitope-Based DNA Vaccine against Other Approaches

The continuous emergence of antigenically drifted variants of seasonal influenza viruses and the threat of antigenically different pandemic influenza viruses raise interest in the development of broadly protective influenza vaccines. This vaccine would be a universal vaccine that can provide cross-protection against different subtype influenza A viruses along with influenza B viruses. These stem epitope-based universal DNA vaccines are based on the broadly cross-reactive neutralizing antibodies directed against an epitope in the highly conserved stem (stalk) of the influenza HA and prevent influenza infection. We can also develop fused DNA vaccine with internal influenza protein or other proteins that induce cellular immune response that does not seem to contribute to preventing infection. It speeds up the elimination of the virus, helps to recover from infection, and also prevents influenza-associated complication. We can also develop multiepitope-based DNA vaccine that can protect against other complications associated with influenza infection such as secondary bacterial infection and may result in better activation of immune response to provide cross-protection against the emerging viral strains [41]. These vaccines are more broadly cross-protective than the currently licensed influenza vaccines and do not need to be administered or updated almost every year. These vaccine productions are not time consuming as compared to the traditional influenza vaccine. They are simply vector-based conserved DNA vaccines that can be delivered intranasally or intramuscularly and in the antigen presenting cell of the host body. The host itself synthesizes and is able to induce both humoral and cellular immune responses against immunogen.

## 7. Limitation of HA2-Based DNA Vaccines

Effective delivery of vector DNA across the plasma membrane, stability, and nuclear targeting are the main challenges in the development of HA2-based DNA vaccine. There are various mechanical, chemical, and electrical based delivery systems, but there is still a need to develop an ideal delivery system that can induce effective immune response to provide cross-protection against influenza infection. There are still many unsolved questions about the decision to update hemagglutinin stem-based vaccine and its efficiency and efficacy in elder people.

There are chances of integration of plasmid DNA vaccine into the genome of immunized host and it is also inefficient in nondividing mature cells such as myocytes. These problems can be overcome by using RNA-based vaccine. mRNA of antigen that encodes epitope can be directly transfected into cytoplasm that results in the high level of expression as compared to the DNA vaccine, but there are some issues in the stability.

## 8. Conclusion

The current influenza vaccine needs to be formulated annually, provides limited protection, and consumes time. Inaccurate prediction in selection of strain for vaccine and only strain-specific or closely related variants protection reduce the efficacy of vaccine. So there is an urgent need to develop a truly universal vaccine that covers all subtypes of influenza A viruses and both lineages of influenza B viruses, thereby providing broad cross-protection and eliminating the need for annual formulations. The HA2 subunit of hemagglutinin (HA) is more conserved than the HA1 subunit and shows cross-protection against different subtype influenza A viruses along with influenza B viruses.

Vector-based DNA vaccine can be used to generate effective HA2 subunit- (stem-) specific immune response. Different combination of fused DNA vaccines can also be constructed that should be able to express various conserved epitope regions of other proteins along with HA2 subunit conserved epitopes. This will increase the efficiency of these stem domain-based universal DNA vaccines against highly diversified influenza viruses and their associated complications. The HA2 subunit-based DNA vaccine can be good candidate for the universal vaccine that may provide cross-protection against heterosubtypes of influenza viruses.
